# Effect of Context on the Contribution of Individual Harmonics to Residue Pitch

**DOI:** 10.1007/s10162-017-0636-6

**Published:** 2017-07-28

**Authors:** Hedwig E. Gockel, Sami Alsindi, Charles Hardy, Robert P. Carlyon

**Affiliations:** MRC Cognition and Brain Sciences Unit, University of Cambridge, 15 Chaucer Road, Cambridge, CB2 7EF UK

**Keywords:** context effects, weights, residue pitch, harmonics

## Abstract

There is evidence that the contribution of a given harmonic in a complex tone to residue pitch is influenced by the accuracy with which the frequency of that harmonic is encoded. The present study investigated whether listeners adjust the weights assigned to individual harmonics based on acquired knowledge of the reliability of the frequency estimates of those harmonics. In a two-interval forced-choice task, seven listeners indicated which of two 12-harmonic complex tones had the higher overall pitch. In context trials (60 % of all trials), the fundamental frequency (F0) was 200 Hz in one interval and 200 + ΔF0 Hz in the other. In different (blocked) conditions, either the 3rd or the 4th harmonic (plus the 7th, 9th, and 12th harmonics), were replaced by narrowband noises that were identical in the two intervals. Feedback was provided. In randomly interspersed test trials (40 % of all trials), the fundamental frequency was 200 + ΔF0/2 Hz in both intervals; in the second interval, either the third or the fourth harmonic was shifted slightly up or down in frequency with equal probability. There were no narrowband noises. Feedback was not provided. The results showed that substitution of a harmonic by noise in context trials reduced the contribution of that harmonic to pitch judgements in the test trials by a small but significant amount. This is consistent with the notion that listeners give smaller weight to a harmonic or frequency region when they have learned that this frequency region does not provide reliable information for a given task.

## INTRODUCTION

In the vision literature, there are many studies examining strategies that observers use to combine information provided by each of multiple cues in a visual environment. It has been shown that the weights applied to different cues are adjusted by the observers according to their reliability: cues that are informative for a task and stable cues that are less often manipulated by the experimenter, receive higher weights than less informative, and less stable cues that are manipulated more often (Jacobs 2002; Triesch et al. 2002). For example, Jacobs and Fine (1999) investigated the relative contribution of motion and texture cues to the perceived depth of a simulated object. After some training, in which one cue was informative and the other was irrelevant, observers’ weights for the relevant cue were larger than for the irrelevant one.

In the auditory domain, flexibility of cue weighting has been studied mainly in the context of speech perception where multiple cues may be used to assign a phonetic category to a given speech sound (see, e.g., Clayards et al. 2008; Idemaru and Holt 2011; Liu and Holt 2015). Mostly, listeners seem to rapidly learn and adjust weights assigned to different cues according to how informative they are (Clayards et al. 2008; Idemaru and Holt 2011; Liu and Holt 2015), but a lingering influence of sensitivity to long-term regularities has also been reported (Idemaru and Holt 2011). Research into cue weighting of non-speech stimuli, for example, discrimination of multitone patterns (Berg 1990; Lutfi and Jesteadt 2006), source identification of impact sounds (Lutfi and Liu 2007), discrimination of force of impact (Lutfi et al. 2011), or discrimination of the level of segments of sounds with an unpredictable temporal profile (Joosten et al. 2016), has shown that subjects do not always weight cues optimally according to how informative they are. Sometimes feedback helps to improve the use of cues (Joosten et al. 2016), but sometimes it does not (Berg 1990; Lutfi and Jesteadt 2006; Lutfi et al. 2011).

The present study investigated whether listeners show adaptive experience-dependent behavior in judging the residue pitch of complex tones. The pitch of complex tones containing many harmonics is usually determined primarily by harmonics falling in a relatively restricted frequency region called the “dominant region” (Ritsma 1967), and dominant harmonics are usually thought to be well resolved in the auditory system (Plomp 1967; but see Jackson and Moore 2013). However, within this dominant region, the exact distribution of dominance can vary markedly between individuals (Moore et al. 1985). Moore et al. (1984, 1985) found that partials with low frequency difference limens (FDLs) contributed more to residue pitch than partials with high FDLs; the relative weights of individual partials to the residue pitch differed across subjects and were negatively correlated with their FDLs. Furthermore, Gockel et al. (2005a) showed that the dominant region for a complex tone of very short duration was shifted upwards in frequency relative to that for the same complex tone with a longer duration (see also Gockel et al. 2007). This shift was predicted based on the well-established finding that the increase in FDL with decreasing stimulus duration is more marked at low than at high frequencies (see, e.g., Liang and Chistovich 1961; Moore 1973b). The studies of Moore et al. (1985) and Gockel et al. (2005a, 2007) provide evidence that the contribution of a given harmonic to residue pitch is influenced by the accuracy with which the frequency of that harmonic is encoded. This resembles reports of nearly optimal integration of *available* information from studies in other sensory modalities (e.g., Ernst and Banks 2002; Alais and Burr 2004), where the available information was manipulated by corrupting the stimulus in the actual test trials. In contrast, here we investigated whether the relative contribution of individual harmonics to residue pitch would also be affected by experimentally manipulated *experience*. Importantly, the experience, i.e., the acquired knowledge of the listeners, was based on context trials in which the reliability of frequency estimates of individual harmonics was manipulated; the stimuli in the test trials were physically identical in the two different context conditions.

Current models of pitch perception (Meddis and O'Mard 1997; Cariani and Delgutte 1996; de Cheveigné 1998; Balaguer-Ballester et al. 2009) implicitly assume the weighting of individual harmonics in determining residue pitch to be fixed; experience from context trials is not taken into account. Therefore, these models would not predict any effect of context.

## METHODS

### Experimental Design

**FIG. 1:**
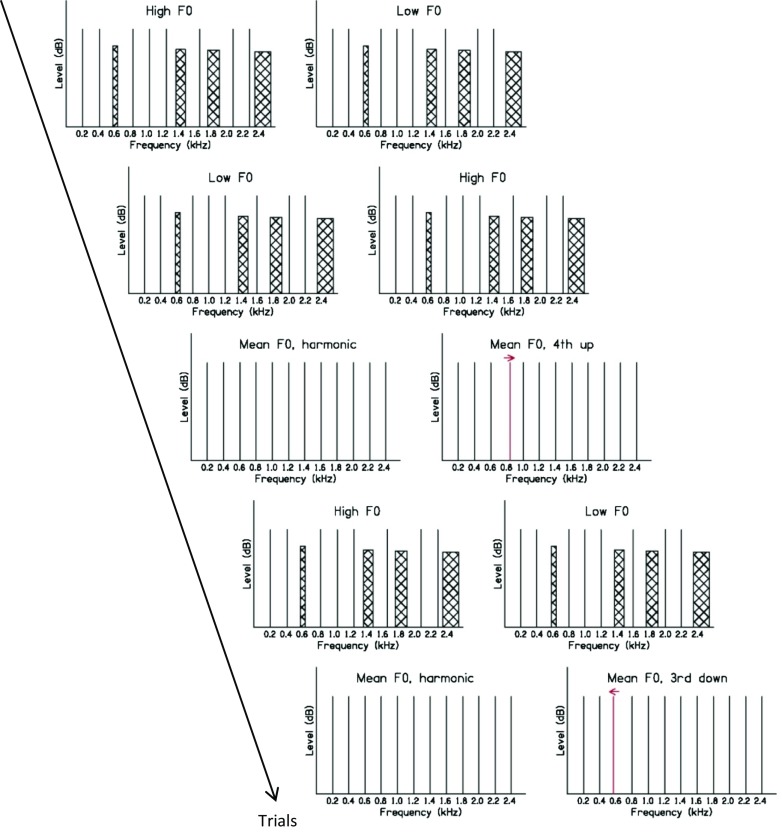
Schematic diagram of a possible trial sequence for condition UNREL3. In context trials, harmonic 3 plus harmonics 7, 9, and 12 are replaced by non-informative noise bands. The difference in F0 between the low and the high cases can be most easily seen by comparing the positions of the high harmonics to the fixed position of the noise bands. Trial 1: context trial with higher F0 in first interval. Trial 2: context trial with higher F0 in second interval. Trial 3: test trial with identical F0s and fourth harmonic shifted upward in frequency. Trial 4: context trial with higher F0 in first interval. Trial 5: test trial with identical F0s and third harmonic shifted downward in frequency.

The stimuli are schematically illustrated in Figure 1. The task was the same throughout the experiment. In a two-interval two-alternative forced-choice task, the listener was asked to indicate which of two harmonic complex tones had the higher overall pitch (residue pitch), ignoring individual components that might pop out. There were two conditions, which were tested in a blocked design. The two conditions differed only with regard to the context trials in which slightly different stimuli were presented in the two conditions (see below and Fig. 1); one condition was designed so that listeners experienced the third harmonic as non-informative (“unreliable”) in the context trials (condition “UNREL3”) and the other condition was designed so that the fourth harmonic was non-informative in the context trials (condition “UNREL4”). In the test trials, the same stimuli were presented in the two conditions. In test trials, either the third or the fourth harmonic was slightly mistuned upwards or downwards in the second interval, while all other harmonics were identical across the two intervals. Moore et al. (1985) showed that a slight mistuning of an individual harmonic in a complex tone leads to a small shift in the residue pitch of the complex; the residue pitch goes up when the frequency of the mistuned harmonic is increased and goes down when it is decreased from its nominal value. In context trials, which made up 60 % of all trials, feedback was provided. In test trials (40 % of all trials), no feedback was provided. The test trials were randomly interspersed with the context trials.

Performance in the test trials was compared across the two conditions to assess whether experience of the different contexts would affect pitch judgements. If the relative contribution of a given harmonic to the residue pitch were affected by its experienced reliability in the context trials, the contribution of the third harmonic to the residue pitch should be smaller in test trials in condition UNREL3 than in condition UNREL4, and therefore the pitch judgement should correctly follow the higher frequency less often in condition UNREL3 than in condition UNREL4. Conversely, the contribution of the fourth harmonic to the residue pitch should be smaller in test trials in condition UNREL4 than in condition UNREL3, and therefore the pitch judgement should correctly follow the higher frequency less often in condition UNREL4 than in condition UNREL3. Thus, for both harmonics, performance in the test trials should be worse in the corresponding unreliable than in the corresponding reliable context condition. Broadly similar approaches of making one cue non-informative during training (trials with feedback) and checking on the effect of this manipulation in test trials (without feedback) where the cue is informative have been used before in non-auditory modalities (e.g., Jacobs and Fine 1999).

### Stimuli

The stimuli were derived from a 200-ms harmonic complex tone with nominal F0 of 200 Hz, containing harmonics 1 to 12. The components had random starting phases (selected afresh for each stimulus presentation) and each component was presented at 50 dB sound pressure level (SPL). In context trials, one randomly chosen interval contained a complex tone with F0 = 200 Hz, while the other interval contained a complex with F0 = 200 Hz + ΔF0. In condition UNREL3, the 3rd harmonic (and the 7th, 9th, and 12th harmonics) was replaced by a noise band (see below) of equal rms level (50 dB SPL); the overall width of each noise band was 10 % of the nominal component frequency (NCF). The NCFs were identical in the two intervals; they were equal to *r* times (200 Hz + ΔF0/2﻿), where *r* corresponds to the harmonic rank of the component. In condition UNREL4, stimuli were the same as in UNREL3 except that the fourth instead of the third harmonic was replaced by a noise band. Thus, in context trials, either the 3rd or the 4th harmonic (plus the 7th, 9th, and 12th harmonics) was non-informative for the task at hand and, as feedback was provided in the context trials, listeners might learn to reduce the weighting of information from these non-informative components/frequency regions. Noise bands rather than sine tones were used to create non-informative components because the pitch of a noise band with center frequency *f* is less clear than that of a sine tone with frequency *f* and discrimination of noise bands with center frequencies around *f* is poorer than discrimination of sine tones with frequencies around *f* (Moore 1973a). The 7th, 9th, and 12th harmonics were replaced by noise bands in addition to the “target component” (either the 3rd or the 4th) in order to prevent perceptual segregation of that component. The test trials were identical in the two context conditions. For these, the complex tones in the two intervals both had an F0 of 200 Hz + ΔF0/2 , i.e., their F0 was halfway between the low and the high F0s of the two stimuli in the context trials, and no harmonic was substituted by a noise band. The complex tone in the first interval was perfectly harmonic. The complex in the second interval had randomly and with equal probability either its third or its fourth harmonic mistuned by a small amount (randomly either up or down in frequency with equal probability). Figure 1 shows a schematic of a possible trial sequence for condition denoted UNREL3, in which the third component was uninformative in the context trials. The percentages of mistuning of harmonics 3 and 4 and ΔF0 were determined individually for each listener in a preliminary experiment (see below).

All stimuli were generated digitally in MATLAB (The Mathworks, Natick, MA). The stimulus duration was 200 ms, including 40-ms raised-cosine onset and offset ramps. The silent interval between the two intervals within a trial was 500 ms. The stimuli for the context trials containing the noise bands were generated in advance of a block of trials (105 trials). Ten exemplars with four independent noise bands and random starting phases of the remaining components were generated for each of the two F0s, and one of each was chosen at random in each context trial. Each noise band was digitally synthesized by summing sinusoids spaced at 1-Hz intervals over the required frequency range (±5 % of the NCF). The amplitudes were drawn from a Rayleigh distribution, and the phases from a uniform distribution in the range 0–360°. The stimuli were played out using a 16-bit digital-to-analog converter (CED 1401 plus), with a sampling rate of 20 kHz, and passed through an antialiasing filter (Kemo VBF25.01) with a cutoff frequency of 7.5 kHz (slope of 100 dB/octave). The overall level was controlled by Tucker-Davis Technologies (TDT) PA4 attenuators. Stimuli were mixed with a continuous background pink noise (low-pass filtered at 5 kHz, Kemo VBF25.03 filter, slope of 48 dB/octave) with a spectrum level of 13 dB (re 20 μPa) at 1 kHz and passed through a headphone buffer (TDT HB7) before being presented binaurally through Sennheiser HD 650 headphones. Subjects were seated individually in an IAC double-walled sound-attenuating booth.

### Experimental Procedure

Each listener was tested in four sessions each lasting for about 2 h (including breaks). In each session, the two context conditions were run in a blocked design. The first half of the session was dedicated to one context condition and the second half of the session to the other. The order of the context conditions was counterbalanced across sessions and listeners. Within each condition, trials were arranged in blocks of 105. Each block started with five context trials to allow listeners to “tune in” to the F0. After that, context trials and test trials were randomly interspersed with the constraint that each set of 10 trials contained 6 context trials and one of each type of the test trials (third mistuned upwards, third mistuned downwards, fourth mistuned upwards, fourth mistuned downwards). Overall, in the main experiment, there were 320 trials for each mistuned harmonic for each direction of mistuning and listener (and 1920 context trials for each context condition).

### Listeners

Seven listeners (mean age = 26 years; range = 21–34 years; 4 females) participated. One of them was the third author. For all listeners, pure tone thresholds were below 20 dB HL at octave frequencies from 250 to 4000 Hz for both ears. At 6000 Hz, pure tone thresholds were below 20 dB HL for both ears for all except one listener, for whom it was at 25 dB HL for one ear. All listeners had some degree of musical training. The seven listeners were selected from a pool of 17 listeners who were initially screened, because for them the third and the fourth harmonics contributed about equally to the residue pitch (in the absence of noise bands). Informed consent was obtained from all listeners. This study was carried out in accordance with the UK regulations governing biomedical research and was approved by the Cambridge Psychology Research Ethics Committee.

### Analyses

For the test trials, discrimination performance in terms of the detectability index *d*′ was calculated separately for each of the two harmonics (third or fourth mistuned) for each listener and context condition. If the component was mistuned upwards and the listener selected interval 2, this was defined as a hit; if s/he selected interval 1, this was defined as a miss. If the component was mistuned downwards and the listener selected interval 1, this was defined as a correct rejection; if s/he selected interval 2, this was defined as a false alarm. Following Macmillan and Creelman (1991), the first stimulus was treated as a standard, and *d*′ was determined as *z*(proportion of hits) minus *z*(proportion of false alarms), where *z*(*p*) is the inverse of the cumulative Gaussian distribution function. Each *d*′ calculation was based on 640 trials in total. Performance for the context trials was also calculated in the usual way for a 2I-2AFC task, i.e., *d*′ = [*z*(proportion of hits) − *z*(proportion of false alarms)] / √2, but was not the main interest of this study.

### Preliminary Experiment

In a preliminary experiment of between 5 and 8 2–h sessions per listener, the following stimulus parameters were tested and determined individually for each listener. Firstly, ΔF0 was chosen so as to give medium to high performance in context trials. Secondly, the percentage of mistuning of harmonics 3 and 4 was chosen independently so as to give low (but above chance) to medium performance in test trials that were interspersed within context blocks providing reliable frequency information for the given harmonic; above chance performance was required to allow measurement of a possible deterioration in the unreliable context condition, and small amounts of mistuning giving low to medium performance rather than large amounts of mistuning were desirable to prevent perceptual segregation of the mistuned harmonic. The final stimulus parameters that were used in the main experiment are shown in Table 1 for each listener. ΔF0 in the context trials was between 1 and 1.5 %, while the amount of mistuning in the test trials was between 0.3 and 2.5 %.

**TABLE 1:** Stimulus parameters used in the main experiment for individual listeners

Listener	Context trials: ΔF0 [%]	Test trials: mistuning of 3rd harmonic [%]	Test trials: mistuning of 4th harmonic [%]
1	1.0	2.5	1.5
2	1.5	1.5	2.0
3	1.0	1.5	1.0
4	1.0	1.0	1.25
5	1.0	0.75	0.75
6	1.5	0.6	0.5
7	1.0	0.6	0.3

## RESULTS

For completeness, and to give an impression of the overall performance resulting from the chosen parameters, Table 2 shows *d*′ scores for each listener averaged across the two context conditions for the context trials and the test trials with either the third or the fourth harmonic mistuned. As intended, *d*′ was (i) quite high in the context trials, except for listener 3, (ii) generally lower in the test trials than in the context trials, and (iii) roughly equal for test trials with the third and with the fourth harmonic mistuned. It is worth noting that *d*′ was significantly higher in the context trials than in the test trials (two-tailed *t* tests, *df* = 6, *p* < 0.005 for context versus third harmonic mistuned and for context versus fourth harmonic mistuned), even though the shift in frequencies of individual harmonics was similar in the context trials and in the test trials (mean across subjects for context trials = 1.14 % and for test trials = 1.13 %). This is consistent with subjects combining information across harmonics to make pitch judgements in the context trials.

**TABLE 2:** Performance, *d*′, averaged across the two context conditions for individual listeners

Listener	Context trials	Test trials: 3rd harmonic mistuned	Test trials: 4th harmonic mistuned
1	3.44	2.18	2.08
2	3.59	2.52	1.96
3	1.85	1.49	1.54
4	2.71	1.89	2.07
5	4.22	2.42	2.31
6	2.61	1.36	2.00
7	3.20	1.15	1.16

**FIG. 2:**
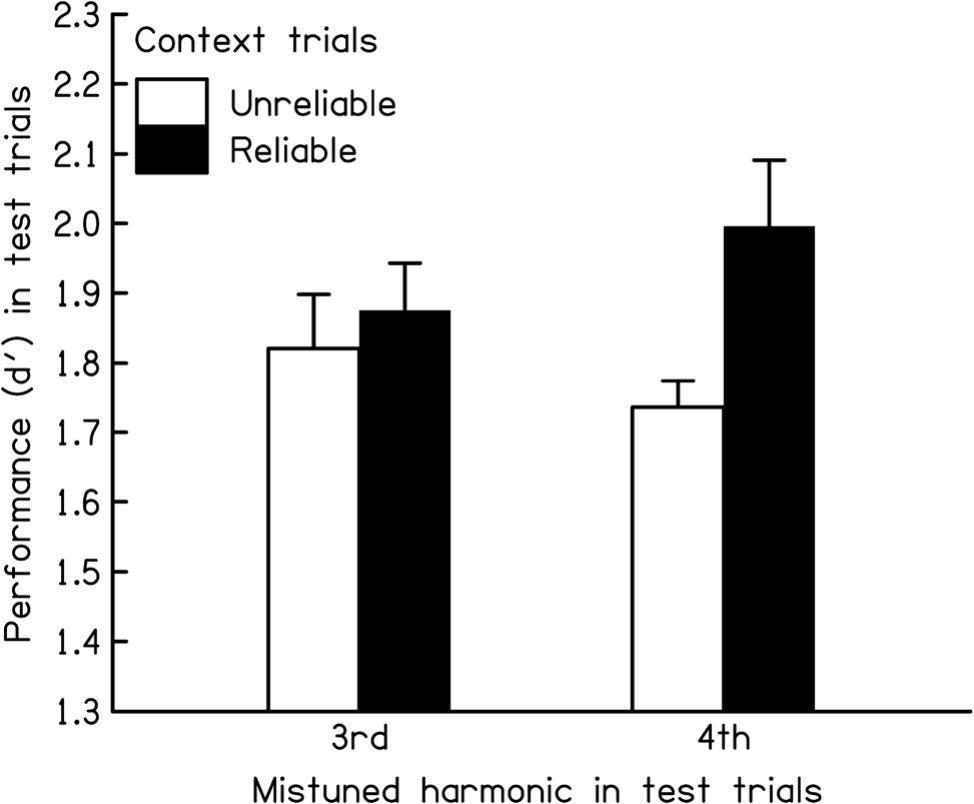
Mean *d*′ values (and standard errors) across the seven subjects in test trials with the third (*left two bars*) and with the fourth (*right two bars*) harmonic mistuned in the unreliable (*white bars*) and reliable (*black bar*s) context conditions. The standard errors are based on normalized data—to equate the mean performance across listeners—as inter-subject variance is irrelevant for within-subjects designs (Loftus and Masson 1994).

The main question was whether performance in the test trials was worse in the unreliable than in the reliable context condition. Figure 2 shows the mean *d*′ values (and standard errors across the seven listeners) separately for test trials in which the third harmonic was mistuned (group of two bars on the left) and for test trials in which the fourth harmonic was mistuned (group of two bars on the right). In both cases, *d*′ was lower in the unreliable context condition (white bars) than in the reliable context condition (black bars). This finding was confirmed by the results of a repeated-measures ANOVA which showed a significant main effect of the reliability of the relevant frequency information in the context trials [*F*(1, 6) = 9.28, *p* = 0.023]. There was no significant main effect of mistuned harmonic number [*F*(1, 6) = 0.02, *p* = 0.90] and, even though the effect of reliability was numerically larger for the fourth than for the third harmonic, the interaction between mistuned harmonic number and reliability of the relevant frequency information in the context trials was not significant [*F*(1, 6) = 3.33, *p* = 0.12]. On average, *d*′ was 8.8 % higher when the context was reliable than when it was unreliable (95 % confidence interval for the effect = [1.7 %, 16 %]). Thus, the observed effect on *d*′ was significant but small.

Assuming that subjects compared the residue pitches of the complex tones in the test trials, we estimated how much the relative contribution of the mistuned harmonic to the residue pitch (its weight) would need to change in order to produce the observed effect on *d*′. We assumed that *d*′ is a linear function of the difference in residue pitch (estimated F0), as Plack and Carlyon (1995) reported *d*′ for F0 discrimination to be roughly a linear function of F0 difference; a given proportional change in estimated F0, therefore, corresponds approximately to the same proportional change in *d*′. A linear relationship between frequency and *d*′ was also reported for pure tone frequency discrimination (Jesteadt and Sims 1975; Turner and Nelson 1982). The calculation of residue pitch for the present stimuli was based on Goldstein’s (1973) model, which assumes that the pitch of a harmonic complex tone can be estimated from the frequencies of the individual harmonics and that the weight of each harmonic is inversely proportional to the variance of the frequency estimate of that harmonic. Specifically, we used Eq. 6 of Gockel et al. (2007), which is based on Goldstein’s model and that specifies the change in the estimated F0 expressed as a proportion of the F0, i.e., the change in the residue pitch of a complex tone, that results from mistuning a single harmonic as:

1 $$ \frac{\overline{\varDelta F0}}{\mathrm{F}0}=\frac{P_j{j}^2}{\sigma_j^2}/\sum_{k=1}^N\frac{k^2}{\sigma_k^2} $$

where *P*_*j*_ is the frequency shift of harmonic *j* (with *j* = 3 or *j* = 4 here) as a proportion of the nominal frequency of the harmonic, *N* is the overall number of components that contribute to the F0 estimate, *k* is harmonic number, and ${\sigma}_k^2$ is the variance associated with the frequency estimate of harmonic *k*. By changing ${\sigma}_j^2$ relative to ${\sigma}_k^2$ (for *k* ≠ *j*), the weight of the mistuned harmonic relative to the weights of the other harmonics is changed. Comparison of different values for *N* and relative weights showed that the change in estimated F0 produced by the frequency shift is roughly proportional to the weight of the shifted harmonic. In other words, a reduction in the weight of the mistuned harmonic by a factor of 2 reduces the pitch shift by roughly 50 %. Therefore, the effect of context on *d*′ that was observed here corresponds to a similar sized effect of context on the actual weights.

## DISCUSSION

### Analytic Versus Synthetic Listening

When a harmonic in a complex tone was replaced by a non-informative noise band in the context trials, pitch judgements in the test trials followed the direction of the frequency shift of that harmonic significantly less often than when a different harmonic was replaced by a non-informative noise band in the context trials. This is consistent with the notion that (i) the replacement of a harmonic by a non-informative noise band in the context trials reduced the contribution of that harmonic to the residue pitch in the test trials and (ii) listeners gave smaller weights to a harmonic or frequency region when they had learned that this frequency region did not provide reliable information for the task. According to this view, the present finding is broadly similar to effects of reliability of information demonstrated in the visual domain, e.g., on the use of different depth cues.

Next, we need to discuss the possibility of a different interpretation. Throughout the experiment, listeners were asked to judge the overall pitch of the sounds and to ignore individual components that might pop out, i.e., listeners were encouraged to use a holistic rather than an analytical listening strategy. While we argue below that it is unlikely that listeners used an analytical listening strategy, we first consider whether, in principle, analytic listening could account for the present results. If, in test trials, listeners compared the individual mistuned component with the corresponding harmonic in the first interval, would performance be expected to deteriorate when that harmonic was substituted by a noise band in the context trials? The answer to this question is not obvious. If the noise band in the target frequency region popped out and led to increased segregation of the mistuned harmonic in the test trials, one might expect performance to improve rather than worsen. On the other hand, if subjects tried to focus on the frequency region of the mistuned harmonic, the change from a mistuned tonal component to a noise might be distracting and might make it harder to tune into the specific target pitch.

The question of the use of analytical versus holistic listening strategies when judging the pitch of a complex tone with a mistuned component is not specific to the “discrimination paradigm” used here but has been discussed before in the context of pitch *matching* paradigms (Gockel et al. 2005b, 2009). However, in the studies of Gockel et al. (2005b, 2009) and Darwin and Ciocca (1992), the mistuned component was presented asynchronously with the remainder of the complex and therefore could always be heard as a separate tone, while in the present study, all components started and stopped together, thus reducing the cues for perceptual segregation. To avoid the possibility that listeners use an analytical listening strategy and compare the individual mistuned component with the corresponding harmonic in the first interval, ideally the two complexes would have no harmonics in common in the two intervals. However, Moore and Glasberg (1990) showed that the differences in timbre between complex tones with no harmonics in common markedly impaired pitch discrimination. Therefore, this approach would have been unsuitable for the present research question, as pitch shifts due to mistuning of a single harmonic are small in comparison to the difference limens for F0 measured for complex tones with no harmonics in common (Moore et al. 1985; Moore and Glasberg 1990). Instead, the present experiment was designed so as to prevent the perceptual segregation of the target component from the remaining components and thus make it less likely that the mistuned component was compared with the corresponding harmonic component. To achieve this, first, in the context trials, listeners needed to “tune in” to the F0 to perform the task optimally, and reliable and informative cues for the task were conveyed only by those harmonics that were not replaced by noise bands. As noted in Results Sect, the superior performance in the context trials compared to the test trials suggests that subjects were at least combining information across harmonics in the context trials, even though the data do not prove that by doing so they were calculating a residue pitch. Second, in the test trials, the first interval always contained the harmonic complex tone; the mistuned harmonic that might pop out to a certain degree (Moore et al. 1986) only appeared in the second interval. Third, the amount of mistuning employed was small. Moore et al. (1986) measured thresholds for hearing out an individual mistuned harmonic as a separate tone from the remainder of the complex. For a 420-ms 200-Hz F0 complex tone, the amount of mistuning required was on average 1.3 and 1.8 % for the third and the fourth harmonics, respectively. For shorter durations, thresholds increased. In the present experiment, the tone duration was 200 ms and the amount of mistuning employed (see Table 1) in most cases was below the thresholds determined by Moore et al. (1986). Also note that listeners 6 and 7, for whom the amount of mistuning employed here was smallest (0.3–0.6 %), showed the same effect of context reliability as was visible in the mean results.

One additional possible factor arises from evidence for the existence of frequency shift detectors (“FSDs”) in the auditory system (Demany and Ramos 2005), and that these FSDs may lead to the phenomenon of “frequency enhancement (FE)” (Erviti et al. 2011; Demany et al. 2013). Evidence for FSDs comes from a paradigm where, following an inharmonic complex tone, listeners are presented with a probe tone, which, in one task, can be slightly (typically one semitone) higher or lower than one of the components in the complex; subjects are required to report whether this shift is up or down. Demany and Ramos (2005) reported that performance in this task was better than in another task, where the probe tone frequency could equal that of one component in the complex (“present”) or fell mid-way between two components (“absent”). They attributed this to FSDs that were most sensitive to shifts of about one semitone (Demany et al. 2009) and concluded that subjects could hear a shift in the pitch of a component that was not heard in the complex and was not heard retrospectively when the probe was presented. In our test trials, one of the harmonics (third or fourth) differed slightly between the first and second intervals, and so this shift may have been detected by an FSD. Subsequently, Erviti et al. (2011) argued that detection of shifts via FSDs could lead to FE, as demonstrated in an experiment where a “test” complex was preceded by a precursor that was identical to the test complex or differed from it (only) in a one-semitone shift in one of its components. A probe tone, presented after the test, had a frequency equal to the possibly shifted component or mid-way between two adjacent components, and subjects reported whether the probe was present in the test complex. Erviti et al. (2011) reported that performance was better when the frequency shift was present rather than absent and concluded that the frequency shift “enhanced” the auditory representation of that component in the test complex, thereby improving performance on the present/absent task. This is relevant to our paradigm because if this improved performance reflects increased segregation of that component, then segregation of the mistuned component in the second interval of our test trials may have been increased by the complex presented in the first interval. This in turn may have allowed subjects to “hear out” the mistuned component with smaller mistunings than in the Moore et al. (1986) study, where the mistuned harmonic complex was not preceded by a harmonic precursor.

Although we cannot completely rule out any influence of FSDs and FEs, several factors reduce the likelihood that they can account for our results. First, the stimulus parameters used to study FSDs and FE are different from those used here. FSDs are most sensitive to shifts of about 7 %, and the smallest shift for which they have been studied is 3 % (Demany et al. 2009)—larger than any mistuning used in our experiment (see Table 1). FSDs and FE have both been studied only using inharmonic complexes, and we do not know how they would be affected by the perceptual fusion that occurs between harmonics (and near-harmonics) of a common fundamental frequency. Second, because FSDs do not lead to the “conscious perception” of the shifted component in the first sound presented (Demany and Ramos 2005), then, even if a FSD caused the mistuned component to “pop out” in the second interval of a test trial, it would not do so in the first interval. Given that subjects were instructed to focus on the residue pitch and that attempts to match the segregated component to the pitch of a fused harmonic complex in the first interval would be unsuccessful, it seems unlikely that subjects would—at least initially—adopt this strategy. Hence for this type of segregation to influence performance, it must be sufficiently salient to have a knock-on effect on subsequent test trials, perhaps by alerting subjects to the possibility of a segregated component and encouraging them to adopt an analytic listening strategy. This would in turn have to survive the presence of intervening context trials on which optimal performance should arise by combining information across harmonics and where the harmonicity and common onset and offset of the components should promote fusion. Indeed, the only physical aspect of the context stimuli that might promote segregation was that the “unreliable” harmonic (as well as three others) was replaced by a noise. As argued above, segregation of that harmonic would likely improve rather than degrade performance. Overall, then, we believe our results are most consistent with the idea that context effects observed here are likely—at least partly—due to a reduction of the contribution of a given harmonic to the residue pitch when it is experienced as non-informative in context trials.

### Context Effects in Hearing

The results reported here suggest that the relative contribution of different harmonics to the perception of pitch is not fully hard wired but is—to a certain degree—plastic. While the effect of context reported here is small, it is consistent with previous reports showing that the relative contribution of individual harmonics to residue pitch depends on duration (Gockel et al. 2005a, 2007). The relative precision of the internal representation of the frequencies of different harmonics changes with duration, and it appears that each harmonic is weighted according to the precision of that internal representation.

Other context effects in relation to pitch perception have been reported. Chambers and Pressnitzer (2014) used Shepard tones (Shepard 1964) separated by a tritone and showed that the perception of the direction of a pitch change from one complex tone to the next could be strongly influenced by the recent history of tones heard. Houtgast (1976) showed that a single harmonic could give rise to the perception of a subharmonic low pitch when it was preceded by a complex tone of similar pitch with many harmonics and both were presented in a noise background at a low signal-to-noise ratio (but see Burns and Houtsma 1999). Effects of pitch priming on the salience of pitch have also been reported. Presentation of a tone with a salient pitch indicating “what to listen for” can improve the perceptual representation and/or the discrimination of high pass filtered iterated rippled noises whose pitch is weak (Butler and Trainor 2013). Also, when discriminating between the frequencies of a very short or a noise-like tone and a longer tone with a more salient pitch, performance is better when the longer tone with the more salient pitch is presented first rather than second in the sequence (Demany et al. 2016).

In Introduction Sect, we mentioned that, in the auditory domain, flexibility of cue weighting has been studied mainly in the context of speech perception and phonetic categories. One exception is the study of Holt and Lotto (2006). They investigated the effect of short-term experience on relative cue weighting in relation to the formation of non-speech categories. They trained listeners to categorize sounds as belonging to one or the other of two previously unknown categories (buttons). The sounds were frequency-modulated sinusoids that differed in center frequency (CF) and modulation frequency (MF). One category contained tones with CFs that were, on average, lower than the CFs for the other category and with MFs that were, on average, higher than that for the other category. The tones were equally spaced in terms of just-noticeable-differences in both the CF and the MF dimensions. Listeners heard one tone at a time and learned category labels through visual feedback (a light). After training (less than 2 h), listeners had to categorize novel stimuli from the same two-dimensional (CF, MF) space (without feedback). Listeners gave more weight to the CF dimension than to the MF dimension when both dimensions were equally informative with regard to category membership. Decreasing the difference between the means of the two CF ranges, leading to more overlap of the two categories on the CF dimension, but keeping other statistics characterizing the distributions constant, did not change the relative weighting of the CF and MF dimensions by the listeners. However, when the variability of the CF was increased and that of the MF decreased, listeners gave more weight to the MF than the CF dimension. Therefore, even when cues were equally informative and discriminable, they were not weighted equally; listeners had a clear “bias” towards the CF cue. However, a change in weighting strategy could be produced by changes in the distribution of the input parameters. It should be noted that the combination of higher CFs with lower MFs and lower CFs with higher MFs might be considered as somewhat unnatural, as it would rarely be encountered.

Effects of the reliability of a *single* cue on (speech) categories have also been demonstrated. Clayards et al. (2008) investigated the effect of the reliability of voice onset time (VOT), which is the primary acoustic cue for voicing of word initial stop consonants (Francis et al. 2008). Listeners identified isolated words as either (i) “beach” or “peach,” (ii) “beak” or “peak,” and (iii) “bees” or “peas.” For each pair, a continuum of VOT values was generated. Short VOTs correspond to words such as “beach” and long VOTs to words such as “peach.” For each word in each pair, the VOT values were drawn from a “Gaussian distribution” with a mean corresponding to the prototypical value for that word in American English (e.g., 0 and 50 ms for beach and peach). Stimuli were synthesized using the Klatt synthesizer (Klatt 1980) with all parameters except VOT held constant for each pair and modeled on natural stimuli. The independent variable was the variance of the two VOT distributions. One group of listeners was presented with exemplars of words generated using a small variance for the two VOT distributions, giving high reliability of the VOT cue, and the other group was presented with exemplars of words generated using a large variance, giving low reliability of the VOT cue. The probability of categorizing a word as, for example, peach as a function of VOT (i.e. the categorization function), was affected by the variance of the two VOT distributions; the slope of the categorization function was shallower for the listeners presented with the VOTs from the two wider distributions than for the group presented with VOTs from the two narrower distributions. Thus, listeners were sensitive to the entire probability distribution of the VOT, and the categorization of words with given VOTs was dependent on the distribution of previously experienced VOTs, i.e., on the experienced reliability of the VOTs.

There is also a long history of research into changes in the perception of a single cue or characteristic of a target sound, for example perceived laterality, following either an adaptor with a fixed parameter value of that same cue or an adaptor with variable and changing parameter values. For example, Dahmen et al. (2010) investigated how auditory spatial processing adapts to stimulus statistics by presenting noise sequences with rapidly fluctuating interaural level differences (ILD) to humans and ferrets. For humans, the mean of the ILD distribution biased the perceived laterality of a following target stimulus, while spatial sensitivity decreased as the distribution’s variance increased. Corresponding neural changes were observed in the inferior colliculus of ferrets; neurons’ ILD preferences adjusted towards the mean of the stimulus distribution and the slope of their rate-ILD functions decreased as the stimulus variance increased. The large body of research into adaptation is beyond the scope of this paper, but adaptation-related mechanisms may of course contribute to changes in the relative weights given to multiple cues.

## SUMMARY

The effect of two different contexts on pitch judgements in test trials was investigated. In one context condition, the third harmonic (plus the 7th, 9th, and 12th) of a complex tone with 12 harmonics was replaced by a non-informative noise band in the context trials, while in the other, the fourth harmonic (plus the 7th, 9th, and 12th) was replaced by a non-informative noise band in the context trials. In randomly interleaved test trials, stimuli were identical in the two conditions; there were no noise bands, the fundamental frequency was 200 + ΔF0/2 Hz in both intervals, and randomly either the third or the fourth harmonic was shifted slightly up or down in frequency with equal probability. Feedback was provided in the context trials but not in the test trials. The replacement of a given harmonic by a non-informative noise band in the context trials affected pitch judgements in test trials; the pitch judgement followed the direction of the frequency shift of that harmonic significantly less often than when a different harmonic was replaced by a non-informative noise band in the context trials. The effect was significant but small. The results are consistent with the notion that (i) the replacement of a harmonic by a non-informative noise band in the context trials reduced the contribution of that harmonic to the residue pitch in the test trials and (ii) listeners gave smaller weights to a harmonic or frequency region when they had learned that this frequency region did not provide reliable information for the task. The finding is broadly similar to effects of reliability of information demonstrated in the visual domain. It suggests that, contrary to the assumptions of contemporary pitch models, the perceived pitch can be influenced to a small extent by the learnt reliability of an individual component of a complex sound.
